# Assessment of drug-induced electrolyte disorders in intensive care units: a multicenter observational study

**DOI:** 10.3389/fmed.2024.1343483

**Published:** 2024-06-04

**Authors:** Yunus Emre Ayhan, Enes Emir İlerler, Damla Sosyal, Muhammed Yunus Bektay, Sait Karakurt, Hayrettin Daşkaya, Kazım Karaaslan, Mesut Sancar

**Affiliations:** ^1^Department of Clinical Pharmacy, Prof. Dr. Cemil Taşcıoğlu City Hospital, Istanbul, Türkiye; ^2^Department of Clinical Pharmacy, Marmara University Faculty of Pharmacy, Istanbul, Türkiye; ^3^Department of Clinical Pharmacy, Basaksehir Cam and Sakura City Hospital, Istanbul, Türkiye; ^4^Department of Clinical Pharmacy, Faculty of Pharmacy, Istanbul University-Cerrahpasa, Istanbul, Türkiye; ^5^Department of Clinical Pharmacy, Bezmialem Vakif University Faculty of Pharmacy, Istanbul, Türkiye; ^6^Department of Chest Diseases and Intensive Care, Faculty of Medicine, Marmara University, Istanbul, Türkiye; ^7^Department of Anesthesiology and Reanimation, Faculty of Medicine, Bezmialem Vakif University, Istanbul, Türkiye

**Keywords:** intensive care unit, electrolyte disorder, clinical pharmacist, drug-related problems, patient safety

## Abstract

**Objective:**

Electrolyte disorder (ED) is frequently encountered critically ill patients during admission or admission to the intensive care unit (ICU). This study aimed to determine the frequency of ED encountered in ICU patients to evaluate the relationship of ED with drugs.

**Methods:**

This prospective, multicenter study was conducted in the medical and anesthesiology ICUs of two training and research hospitals and included patients with at least one ED during admission or hospitalization in the ICUs. The relationship between ED and the drug was evaluated by calculating the logistic probabilistic method scale (LPMS) and the expert panel’s evaluation. The correlation between EDs and LPMS was determined using Kendal tau. A binary logistic regression model was preferred in the analysis of factors related to ED. Statistical significance was set as *p* < 0.05.

**Results:**

A total of 117 patients were included in the study. A total of 165 EDs were detected, including at least one in 88 (75.2%) patients. According to the expert panel, 61 (21.7%) of EDs were drug-related, whereas according to the LPMS, 111 (39.6%) (*p* < 0.001). Mortality (50% vs. 13.7%) and mechanical ventilation rates (52.2% vs. 17.2%) were significantly higher in patients with ED (*p* < 0.001). Patients with ED had 8.352 times higher odds of exhibiting mortality (OR: 8.352, %95 CI: 1.598–43.648, *p*: 0.012) and need mechanical ventilation with higher odds of 3.229 (OR: 3.229 95% CI: 0.815–12.787 *p*: 0.045). Patient who required enteral or parenteral feeding were associated with an increased likelihood of exhibiting ED (respectively OR: 30.057, %95 CI: 2.265–398.892, *p*: 0.01, OR: 5.537, %95 CI: 1.406–21.800, *p*: 0.014).

**Conclusion:**

EDs are very common in the ICU. Dysnatremia was detected more commonly in other EDs. It has also been found that patients with ED are more often under mechanical ventilation, have more prolonged hospitalizations, and have higher mortality rates than patients without ED. The suitability of LPMS for assessing ED-drug relationships in the ICU context is questioned.

## 1 Introduction

Electrolytes play a role in many enzymatic and biochemical metabolic and hemostatic functions (1). One of the important problems in maintaining hemostasis in the body is fluid and electrolyte balance (2). Electrolyte disorder (ED) is frequently encountered in critically ill patients during admission or during hospitalization at the intensive care unit (ICU), because of treatment or multiple comorbiditie as a result of their treatment or multiple comorbidities (3).

The prevalence rates at admission to and during hospitalization in the ICU were reported as 18–33% and 11–16% for hyponatremia, 7–9%, and 6–29% for hypernatremia, respectively (4–6). Dysnatremia during ICU admission and stay has been associated with increased hospital mortality rates (5, 7). The prevalence of hypokalemia and hyperkalemia in ICU patients has been reported to be 17.4 and 12%, respectively (8). Two separate meta-analyses associated hypomagnesemia with higher mortality rates, need for mechanical ventilation, and incresead length of ICU stay (9, 10). The prevalence rates of hypocalcemia and hypercalcemia reported in ICU patients are 55–88 and 2%, respectively (11, 12). Hypercalcemia was independently associated with hospital or ICU mortality, mainly because of underlying malignancies (13). Similarly, hypocalcemia was also associated with longer ICU lengths of stay and increased mortality. Hypophosphatemia is common in the ICU, and has been observed in approximately 28% of critically ill patients (14). Hypophosphatemia is associated with longer ICU and hospital stays, increased risk of arrhythmia, and respiratory distress (15).

Diuretics, trimethoprim-sulfamethoxazole, amphotericin B, dexamethasone, ifosfamide, lithium, foscarnet, hypertonic 3% saline, and antibiotics containing sodium (fosfomycin, piperacillin-tazobactam) can be given as examples of drugs that cause hypernatremia (7, 16–18). Salbutamol, loop and thiazide diuretics; glucocorticoids, amphotericin B, and carbonic anhydrase inhibitors causes drug-induced hypokalemia (19, 20). Causes of drug-induced hyperkalemia include penicillin G, spironolactone, amiloride, trimethoprim, and angiotensin-converting enzyme inhibitors (21, 22). Among the drugs that disrupt the calcium balance, such as bisphosphonates, calcitonin, cisplatin, cyclophosphamide, cytarabine, and doxorubicin; phenytoin, phenobarbital, rifampicin, isoniazid, and furosemide can be counted (23). Lithium, Vitamin D, intoxication, and teriparatide are examples of drug-induced hypercalcemia (24). Antacids, sucralfate, phosphate binding agents, and insulin can be counted among the drugs that cause disturbances in phosphate balance (25, 26).

In the literature, studies examining drug-related ED in the adult ICU setting are limited in number. However, a study addressing this issue stated that ED is frequently seen in ICUs. Therefore, it has been attempted to increase clinicians’ awareness about drugs that can potentially cause electrolyte imbalance (27).

This study aimed to determine the frequency of ED encountered in ICU patients, to evaluate its relationship with drugs, and evaluate the applicability of the current logistic probabilistic method scale (LPMS) in the ICU setting. This study aimed to determine the frequency of ED encountered in ICU patients, to evaluate its relationship with drugs, and evaluate the applicability of the current logistic probabilistic method scale (LPMS) in the ICU setting (28).

## 2 Materials and methods

### 2.1 Study design and patients

This observational multicenter prospective study was conducted in the medical and anesthesiology ICUs of two university hospital in Istanbul, Türkiye (Bezmialem Vakif University, and Marmara University) between 01.09.2022 and 01.03.2023. Patients who had at least one ED (hyponatremia, hypernatremia, hypokalemia, hyperkalemia, hypomagnesemia, hypermagnesemia, hypophosphatemia, or hyperphosphatemia) at the time of admission to or during their stay in the ICU were included in the study.

### 2.2 Inclusion and exclusion criteria

Patients aged ≥18 years and hospitalized in the ICU for ≥24 h were included in the study. Patients with missing data and/or who did not use medication during their ICU stay were excluded from the study.

### 2.3 Data collection and evaluation

Patients with ED included in the study were followed daily by a longer ICU stay clinical pharmacist and intensive care physician during their ICU stay. Patient demographic information, drugs used during hospitalization, laboratory data, drug and medical history, acid–base balance, blood gas values, and fluid balance were followed up during the daily evaluations.

The possible causes of ED and its relationship with drugs were evaluated by an expert panel consisting of intensive care specialists (SK and HD) and clinical pharmacist (YEA, MYB), and the current LPMS was used as the assessment material/scale for the relationship between ED and drugs. For this purpose, intensive care specialists’ evaluation was recorded to determine whether the possible cause was drug-related in patients with ED. The current LPMS of each patient with ED was also calculated by a clinical pharmacist independently of the intensive care specialists. Thus, after the intensive care specialist evaluated the relationship between ED and clinical condition, the relationship between ED and drugs was examined. As a result, each expert’s evaluation was recorded independently of each other and finally discussed, and the results were decided.

LPMS evaluates the relationship between a drug and its side effects by focusing on six fundamental parameters: time of onset, exposure and re-exposure, investigation of other etiologies, risk factors for drug interaction, reaction or toxic plasma concentration at the application site or validated laboratory test, and symptomatology. The numerical sum of the statistical weights obtained from the answers to the specified parking meters is converted into probability using the logistic function. According to the *p*-value obtained afterward, the possible relationship between the side effect and the drug is interpreted (28). The evaluation process of EDs is summarized below;

1. At least two consecutive values outside the normal range were considered to be ED.
2. ED was considered to be resolved when at least two consecutive normal values were observed.
3. If the same ED is seen again after the development and resolution of ED in the same patient, it is added to the frequency of occurrence.
4. During the evaluation of the clinical etiology, if the relevant ED was not evaluated, the statements “not evaluated” and “not calculated” were added to the *p*-value of LPMS calculation part. If a result could not be reached in the evaluation made by the intensive care specialist, the statement “decision could not be made” was added.
5. In the absence of any potential drugs that could be associated with the patient’s ED, the *p*-value was stated as “not calculated.”
6. The local drug monograph and UpToDate® (Wolters Kluwer Health Inc., 2023) reference sources were used to evaluate the association of drugs with ED. The Sanford Guide to Antimicrobial Therapy was also used to examine the relationship between antimicrobial agents and ED.

### 2.4 Sample size

The study examined patients who met the inclusion criteria in the ICU during the study period (6 months). For the sample size of the study, it was decided to include a total of 115 patients in the study by adding a 15% dropout rate to the calculation made over the standard deviation 1, alpha 0.05, and 95% power values based on the monthly number of patients hospitalized in the ICU.

### 2.5 Data analysis

As descriptive statistics, mean, median, standard deviation, and interquartile range (IQR) or count and percentages are given for continuous variables. The frequency and percentage are given for categorical variables. The normality of continuous variables was evaluated using the Kolmogorov–Smirnov and Shapiro–Wilk test, histogram analyses, Q-Q plots, skewness and kurtosis analysis of data. It was determined that the data showed a normal distribution. The difference between groups was analyzed using independent *t*-test. Chi-square tests are used to investigate the relationship between categorical variables. The Pearson correlation coefficient was calculated in the correlation analysis. According to the correlation coefficient r value, the relationship was defined as “low” (0.01–0.29), “moderate” (0.30–0.70) and “high” (0.71–0.99). The probability status of different demographic situations and survey scores were determined by the odds ratio. Univariate logistic regression analysis was used to determine which variable(s) are significant by using *p* < 0.20. Significant variables are included in the binary logistic regression analysis. Binary logistic regression analysis was performed, and estimated risk values and confidence intervals were provided. This study was conducted to identify the variables that are useful in predicting the existence of polypharmacy. The Nagelkerke *R* square value was used to assess the model’s explanatory power, and the Hosmer and Lemeshow test was used to assess the model’s fit. The missing data were excluded from the analysis. All the data were statistically analyzed by using Statistical Package for Social Science (SPSS) version 26® and Jamovi version 1.6 software. Univariate and multivariate logistic regression analysis was conducted to identify factors associated with the electrolyte imbalance. Statistical, significance was set as *p* < 0.05.

## 3 Results

A total of 117 out of 165 hospitalized patients were included in the study. Sixty patients (51.3%) were male, the mean age was 63.7 (±16.8), and the number of comorbidities was 2 (±1.75) diseases. The most common reason for ICU admission was respiratory distress (21.9%) (Table 1).

**Table 1:** Demographic characteristics of the study participants.

Variable	
Age, (Mean ± SD)	63.7 ± 16.8
Gender, *n* (%)
Male	60 (51.3)
Female	57 (48.7)
Reasons for admission to the intensive care unit*, *n* (%)
Convulsive seizure	4 (3.2)
Cardiac arrest	5 (4.0)
General condition disorder	7 (5.6)
Sepsis/Septic shock	9 (7.3)
Pneumonia	16 (13.0)
Post-op	21 (17.02)
Respiratory distress	27 (21.9)
Other	34 (27.6)
Comorbidities* *n* (%)
Pulmonary embolism	7 (2.4)
Heart failure	8 (2.7)
Atrial fibrillation	10 (3.4)
Chronic kidney disease	12 (4.1)
Lung cancer	12 (4.1)
Cerebrovascular event	14 (4.8)
Chronic obstructive pulmonary disease	19 (6.6)
Other types of cancer	25 (8.7)
Coronary artery disease	26 (9.0)
Diabetes mellitus	41 (14.3)
Hypertension	51 (17.8)
Other	55 (19.2)
*Admission GCS, (Mean ± SD)*	*13.3 ± 3.06*
*Number of comorbidity, (Mean ± SD)*	*2.8 ± 1.75*
*Length of stay in ICU (days), (Mean ± SD)*	*10.5 ± 9.58*
*Number of drugs in admission, (Mean ± SD)*	*11.11 ± 4.08*
*Number of drugs at discharge, (Mean ± SD)*	*10.2 ± 4.41*
Nutrition type, *n* (%)
Oral	46 (39.3)
Enteral	38 (32.5)
Parenteral	6 (5.1)
Intravenous Dextrose	27 (23.1)
Kidney status, *n* (%)
Normal	67 (57.3)
Acute kidney failure	30 (25.6)
Hemodialysis	4 (3.4)
Continuous renal replacement therapy	16 (13.7)
Mechanical ventilation status, *n* (%)
Yes	51 (43.6%)
Mortality, *n* (%)
Yes	48 (41%)

*Since a patient has more than one reason for admission, this number is higher than the number of patients.

GCS, Glasgow coma scale, ICU, Intensive care unit.

A total of 165 EDs were detected, including at least one in 88 (75.2%) of the patients. The mean number of EDs per patient was 1.4 ± 1.57. Hyperkalemia (19.8%), hypernatremia (16.8%), and hyponatremia (13.8%) were the most common EDs in patients (Table 2).

**Table 2:** Distribution of frequency of electrolyte disorders.

Electrolyte Disorders	Frequency in all ED, *n* (%)	Values of ED, Mean (min - max)	Patient, *n* (%)
Hypernatremia	28 (16.8)	149.05 (145.0–155.0)	26 (22.2)
Hyponatremia	22 (13.8)	131.45 (120.0–135.0)	21 (17.9)
Hyperkalemia	33 (19.8)	6.0 (5.14–7.10)	27 (23.1)
Hypokalemia	14 (8.4)	3.11 (2.30–3.47)	14 (12)
Hyperphosphatemia	8 (4.8)	5.82 (3.80–9.90)	8 (6.8)
Hypophosphatemia	21 (12.6)	1.86 (1.10–2.60)	20 (17.1)
Hypermagnesemia	9 (5.4)	3.0 (2.70–3.41)	8 (6.8)
Hypomagnesemia	18 (10.8)	1.54 (0.94–2.80)	17 (14.5)
Hypercalcemia	1 (0.6)	12.7	2 (1.7)
Hypocalcemia	10 (6.0)	8.5 (7.10–8.70)	9 (7.7)
Total	165 (100)		88 (75.2)

ED, Electrolyte disorder.

The mean day of observation of ED during patients ICU stay was 6 (±7.96). ED lasted an average of 4.5 days (±3.16). According to the expert panel, 21.7% of EDs were drug-related, while according to the LPMS, 39.6% were drug-related. A statistically significant difference was found between the expert panel and LPMS in the rates of possible causes of ED (Table 3). According to the LPMS, the most common causes of drug-related EDs were furosemide (44.1%), sodium chloride 0.9% (11.6%), pantoprazole (9.3%), and amphotericin b (4.6%). According to the LPMS, the clinical conditions causing EDs were intake deficiency (52.9%), renal failure (15.6%), and hypervolemia (7.8%). According to the expert panel, the most common causes of drug-related EDs were furosemide (40%), pantoprazole (20%), and 0.9% sodium chloride (10%). According to the expert panel, the clinical conditions causing EDs were found to be a lack of intake (33.8%), hypovolemia (24.1%), and renal failure (8.0%).

**Table 3:** Comparison of expert panel and LPMS decisions.

Electrolyte disorder	Expert panel decision, *n* (%)	LPMS decision, *n* (%)	*p* value
Drug-induced	61 (21.7)	111 (39.6)	<0.001
Depends on clinical situation	148 (52.8)	102 (36.4)	
Could not be evaluated/decided	71 (25.3)	138 (49.2)	
Total	280 (100)*	280 (100)*	

* More possibilities than the total number of EDs (*n* = 165) were evaluated because more than one possible cause of an ED was evaluated.

LPMS, Logistic probabilistic method scale.

A significant correlation was found between the total number of ED patients and the total number of days of hospitalization in the ICU (*r*: 0.457, *p* < 0.001), the number of drugs at discharge (*r*: 0.245, *p*: 0.019), mechanical ventilation status (*r*:-0.279, *p*: 0.002) and mortality status (*r*:-0.218, *p*: 0.018). No significant correlation was found between the Glasgow coma scale (GCS) score at admission, type of nutrition, renal status, number of drugs on admission, and total number of EDs in the patients. There was a statistically significant difference between the presence of ED in the patients, ICU admission GCS score, number of drugs in hospitalization, mechanical ventilation status, mortality status and total length of stay (Table 4). In addition, patients with ED (11.7 ± 9.1) had a statistically significant longer length of stay in the ICU compared with patients without ED (6.9 ± 10.2) (*p*: 0.019). In addition, mortality (50% vs. 13.7%) and mechanical ventilation rates (52.2% vs. 17.2%) were significantly higher in patients with ED than in those without ED (*p*: 0.001 and *p*: 0.001, respectively).

**Table 4:** Distribution of the relationship between categorical data and the presence of electrolyte disorders.

Variable	Electrolyte disorder status		*p* value
	Yes	No	
Age, *n*
<64	34	9	0.461
≥64	54	20	
Gender, *n*
Male	46	14	0.709
Female	42	15	
Admission GCS, *n*
<14	24	2	0.021
≥14	33	15	
Number of comorbidity, *n*
<3	39	13	0.962
≥3	49	16	
Length of stay in ICU (days), *n*
<11	50	27	<0.001
≥11	38	2	
Number of drugs in admission, *n*
<12	46	24	0.006
≥12	39	5	
Number of drugs at discharge, *n*
<11	50	24	0.810
≥11	12	5	
Nutrition type, *n*
Oral	38	8	0.136
Others (Enteral, Parenteral, IV Dextrose)	50	21	
Kidney status, *n*
Normal	49	18	0.547
Others (Acute kidney failure, Hemodialysis, Continuous renal replacement therapy)	39	11	
Mechanical ventilation status, *n*
Yes	46	5	<0.001
No	42	24	
Mortality, *n*
Yes	44	4	<0.001
No	44	25	

GCS, Glasgow coma scale; ICU, Intensive care unit; IV, Intravenous.

Binomial logistic regression analysis was performed to determine the effects of age, number of comorbidities, ICU stay, nutritional status, number of medications during ICU stay and number of medications during discharge. The logistic regression model was statistically significant, *χ*2(6) = 26.626, *p* < 0.001. The model explained 44.3% (Nagelkerke *R*^2^) of the variance in ED and the model correctly classified 82.9% of cases. Sensitivity was 41.4%, specificity was 96.6%, positive predictive value was 83.3% and negative predictive value was 80.0%. The model reliability was tested with an omnibus ANOVA test for model coefficients (*p*: 0.001) and Hosmer and Lemeshow Test (*p*: 0.970). Of the 8 predictor variables, only three were statistically significant: mortality, mechanical ventilation, and nutritional status (Table 5). Patients with ED had 8.352 times higher odds of exhibiting mortality (%95 CI: 1.598–43.648, *p*: 0.012) and need mechanical ventilation with higher odds of 3.229 (95% CI: 0.815–12.787 *p*: 0.045). Patient who required enteral or parenteral feeding were associated with an increased likelihood of exhibiting ED (respectively OR: 30.057, %95 CI: 2.265–398.892, *p*: 0.01, OR: 5.537, %95 CI: 1.406–21.800, *p*: 0.014).

**Table 5:** Binary logistic regression analysis of factors related with electrolyte disorders.

Variables	Electrolyte disorder					
	Univariate analysis			Multivariate analysis		
	OR	95% CI for Odds ratio	*p* values	OR	95% CI for Odds Ratio	*p* values
Age	1.015	0.98–1.043	0.280			
Comorbidity	0.855	0.655–1.100	0.223			
ICU Stay (days)	0.922	0.858–0.990	0.025	0.953	0.893–1.016	0.142
Nutritional Status		-				
Oral feeding	*Reference*					
Enteral Feeding	9.500	1.478–61.071	0.018	30.057	2.265–398.892	0.010
Parenteral Feeding	3.800	1.296–11.144	0.015	5.537	1.406–21.800	0.014
Number Medication during ICU stay	0.854	0.754–0.969	0.014	0.944	0.801–1.113	0.494
Number of Medication during hospital discharge	0.939	0.852–1.034	0.199	1.090	0.922–1.289	0.313
Mortality						
*No*	*Reference*					
*Yes*	6.250	2.009–19.448	<0.001	8.352	1.598–43.648	0.012
Mechanical Ventilation						
*No*	*Reference*					
*Yes*	5.257	1.839–15.029	<0.001	3.229	0.815–12.787	0.045

ICU: Intensive care Unit.

## 4 Discussion

To the best of our knowledge, this is a one of the first studies in the literature that examines EDs in the ICU and their relationship with the drugs. This study investigates the incidence of EDs in the ICU population and the role of LPMS and expert panel in evaluating the relationship between EDs and drugs. Accordingly, it was observed that EDs are common in the ICU, ED is associated with mortality. The LPMS correlates ED with medication at a higher rate than the expert panel.

Due to the severe condition of patients in the ICU, the variety of drugs used in the treatment, and many factors affecting the patient’s hemodynamic stability, EDs have been widely detected in other studies (5, 6, 27, 29). Studies evaluating all EDs in the ICU are limitted. It has been demonstrated in many studies that dysnatremia is the most common ED (5, 6, 29, 30). In this study, dysnatremia was higher than hyperkalemia and hypokalemia. Sodium and potassium-related EDs are expected in the ICU due to many mechanisms such as acid–base, fluid balance, renal failure, and drugs (6, 29, 30). Less frequent follow-up of other electrolytes, such as magnesium and phosphorus, and later recognition of their symptoms may explain why EDs of these electrolytes are less frequent (14, 27). When the rates of disorders of all electrolytes examined in this study are compared with those in the literature, different rates of ED are observed. This difference may be due to differences in laboratory reference ranges for electrolytes considered in studies, frequency of monitoring, and differences in ICU populations (31).

It was observed in this study that EDs in the ICU were detected mainly in the first week of admission to the ICU and treated in an average of 4–5 days. Stelfox et al. reported the median time of detection of dysnatremia as 2 days (6). It may cause prolonged detection and treatment of ED because of hospitalizations to the ICU for severe and vital reasons. Another possibility is the treatment of EDs after the control and management of other emergencies. The presence of at least 1–2 EDs per patient and ED being statistically significantly associated with mortality require rapid control of EDs (5, 6).

Many factors can cause EDs in the ICU. Among these factors, drugs also have a certain ratio (27). However, no scale or scoring systems has been developed in the literature for associating EDs with drugs in the ICU. Therefore, the use of LPMS, one of the few scales that evaluates the relationship of side effects with the drug, was considered. In this study, EDs were associated with the drug at a higher rate than the expert panel when evaluated using LPMS. According to both the LPMS and the expert panel, the drugs and clinical factors causing EDs were determined to be quite similar. However, the causes of EDs could not be evaluated/decided in approximately half of the patients because of the complicated calculation method of LPMS and its unsuitability for use in the ICU. On the other hand, in the evaluation made with the expert panel, possible causes were revealed depending on the clinical conditions in approximately half of the EDs. However, due to the high rate of undecidable situations with LPMS and the significant proportional difference between the expert panel and LPMS, there is a need for a scale that evaluates the relationship between EDs and drugs, considering the clinical factors of the patients. In this case, the use of LPMS in the ICU is not considered appropriate and practical.

There was a positive correlation between the length of hospital stay and low GCS in ICU patients and a negative correlation between EDs and mechanical ventilation and hospitalizations, resulting in mortality. Based on this information, there is a direct relationship between the worsening of the patient’s prognosis and EDs. On the other hand, in the logistic regression analysis, a significant relationship was revealed with EDs in patients being mechanically ventilated, fed by means other than oral, and hospitalizations resulting in mortality (6, 7, 29, 30, 32). Therefore, it is predicted that the outcomes of the patients will be improved by the diagnosis and management of EDs in patients hospitalized in the ICU, especially in the first week of the patient’s ICU, in a shorter time (5, 30). Long-term stays in the ICU bring many complications due to the nature of the ICU environment. Therefore, long-term ICU hospitalizations, the need for mechanical ventilation, and the patient’s enteral feeding tubes or parenteral nutrition conditions make the management and care of the patient more complicated (32). During this prolonged hospitalization, at ICU, the patient’s acid base, fluid balance, nutritional needs, and the variability of drug treatments significantly affected the ED rates of the patients. EDs are particularly common in mechanically ventilated patients and significantly impact patient outcomes during treatment and weaning from mechanical ventilation. Patients requiring mechanical ventilation are likely to have decreased electrolyte levels. It is stated that unbalanced serum electrolytes and delay in their recovery prolong the duration of mechanical ventilation, stay in the ICU, and are associated with increased comorbidity and mortality (14, 29, 33, 34). For these reasons, it was found in this study, similar to the literature, that patients with ED were statistically significantly more likely to be under mechanical ventilation, with a longer ICU stay, and with higher mortality than patients without ED (5–7, 34).

The limitations of this study are that the relationship of ED with the drug was not controlled using different scales, and only a small number of patients were included in the study. In an environment where there are many factors affecting ED, such as the ICU, other factors, such as fluid, acid–base balance, and renal failure, which affect ED were considered in the expert panel but were not mentioned in this study because they were not the primary aim of the study. Because this was a multicenter study, there was also a difference in the frequency and order of laboratory follow-up of electrolytes in the centers. These differences may have caused inconsistencies in the ED rates. The study gains strength both in Turkey and in the literature in terms of investigating all EDs and the relationship of EDs with drugs in the patient group in the ICU. In addition, this study also included surgical, medical, cardiac, and neurological/traumatic patients without focusing only on a specific ICU patient population. The results of this study are likely to generalize to EDs in other ICU settings.

## 5 Conclusion

In conclusion, EDs are quite common in the ICU. Dysnatremia was detected more commonly than in other EDs. It has also been found that patients with ED are more often under mechanical ventilation, with more prolonged hospitalizations, and have higher mortality rates than patients without ED. In evaluating the relationship between EDs and drugs in the ICU, it was observed that the LPMS associated EDs with the drugs at a higher rate than the expert panel. According to the LPMS and the expert panel, drugs that cause ED have been similarly identified. However, the causes of EDs in approximately half of the patients with LPMS have not been evaluated/decided. Therefore, LPMS does not appear to be a practical and appropriate scale for evaluating the ED-drug relationship in the ICU.

## Data availability statement

The original contributions presented in the study are included in the article/supplementary material, further inquiries can be directed to the corresponding author.

## Ethics statement

The studies involving humans were approved by Ethics Committee of Marmara University. This study was conducted in accordance with the local legislation and institutional requirements. The participants provided their written informed consent to participate in this study.

## Author contributions

YEA: Conceptualization, Data curation, Formal analysis, Investigation, Methodology, Project administration, Resources, Writing – original draft, Writing – review & editing. EEI: Conceptualization, Data curation, Writing – original draft. DS: Data curation, Writing – original draft. MYB: Conceptualization, Data curation, Investigation, Methodology, Project administration, Resources, Supervision, Visualization, Writing – original draft, Writing – review & editing. SK: Conceptualization, Formal analysis, Resources, Supervision, Writing – original draft, Writing – review & editing. HD: Resources, Supervision, Visualization, Writing – review & editing. KK: Visualization, Writing – review & editing. MS: Formal analysis, Project administration, Resources, Supervision, Visualization, Writing – review & editing.
